# Radiomics Analysis of Lymph Nodes with Esophageal Squamous Cell Carcinoma Based on Deep Learning

**DOI:** 10.1155/2022/8534262

**Published:** 2022-09-13

**Authors:** Li Chen, Yi Ouyang, Shuang Liu, Jie Lin, Changhuan Chen, Caixia Zheng, Jianbo Lin, Zhijian Hu, Moliang Qiu

**Affiliations:** ^1^School of Arts and Sciences, Fujian Medical University, Fuzhou, Fujian 350122, China; ^2^Fujian Key Laboratory of Medical Bioinformatics, Fuzhou, Fujian 350122, China; ^3^School of Public Health, Fujian Medical University, Fuzhou, Fujian 350122, China; ^4^College of Mathematics and Informatics, Fujian Normal University, Fuzhou, Fujian 350122, China; ^5^Affiliated Fuzhou First Hospital of Fujian Medical University, Fuzhou, Fujian 350009, China; ^6^The First Affiliated Hospital of Fujian Medical University, Fuzhou, Fujian 350009, China

## Abstract

**Purpose:**

To assess the role of multiple radiomic features of lymph nodes in the preoperative prediction of lymph node metastasis (LNM) in patients with esophageal squamous cell carcinoma (ESCC).

**Methods:**

Three hundred eight patients with pathologically confirmed ESCC were retrospectively enrolled (training cohort, *n* = 216; test cohort, *n* = 92). We extracted 207 handcrafted radiomic features and 1000 deep radiomic features of lymph nodes from their computed tomography (CT) images. The *t*-test and least absolute shrinkage and selection operator (LASSO) were used to reduce the dimensions and select key features. Handcrafted radiomics, deep radiomics, and clinical features were combined to construct models. Models I (handcrafted radiomic features), II (Model I plus deep radiomic features), and III (Model II plus clinical features) were built using three machine learning methods: support vector machine (SVM), adaptive boosting (AdaBoost), and random forest (RF). The best model was compared with the results of two radiologists, and its performance was evaluated in terms of sensitivity, specificity, accuracy, area under the curve (AUC), and receiver operating characteristic (ROC) curve analysis.

**Results:**

No significant differences were observed between cohorts. Ten handcrafted and 12 deep radiomic features were selected from the extracted features (*p* < 0.05). Model III could discriminate between patients with and without LNM better than the diagnostic results of the two radiologists.

**Conclusion:**

The combination of handcrafted radiomic features, deep radiomic features, and clinical features could be used clinically to assess lymph node status in patients with ESCC.

## 1. Introduction

Esophageal cancer (EC) is a common malignant digestive tumor with a high incidence and death rate in China, with esophageal squamous cell carcinoma (ESCC) being the most common [1]. Most patients with EC are diagnosed as locally middle or advanced, and are, therefore, not eligible for surgery at the time of diagnosis, which results in poor prognosis and low survival rate [2, 3]. Lymph node metastasis (LNM) is one of the main factors that affect prognosis because the spread of lymph nodes (LN) is highly variable and unpredictable [4]. As such, accurate assessment of LN status is an important factor in tumor staging and a prerequisite for optimal treatment. The American Joint Committee on Cancer (AJCC) proposed the eighth edition of the International Staging Standard for Esophageal Cancer, including the number of LNM in the postoperative staging of LNs, and provided a clinical staging system for EC based on preoperative imaging for the first time [1].

Computed tomography (CT) is the most commonly used noninvasive image method to evaluate LN status before and after an operation [5]. As such, it is of great significance in the clinical staging of early screening LNs for EC, regional LN dissection, and nonsurgical radiotherapy and chemotherapy. However, at present, the morphological criteria for judging the size of LN based on CT are still controversial, as the size and axial ratio of LN were manually measured by doctors with different clinical diagnosis experience [6]. Therefore, whether LNM is judged or not is still primarily based on postoperative pathological examination results [1]. This has a certain influence on the choice of precise surgical procedures and the clinical stage of chemoradiotherapy [7].

The high-throughput radiometric analysis provides a large amount of medical image information, including size, shape, texture, and other characteristics of tumors or LNs [8–10]. Several studies have suggested that radiomic analysis of tumors could be used to discriminate prognostic biomarkers from ESCC staging to improve subsequent clinical treatment [11–13]. Therefore, radical analysis can help doctors determine the heterogeneity of tumors and LNs and, thus, make accurate clinical treatment decisions for patients with EC. The identification of LNM using CT radiomics has important clinical significance in LN staging, chemoradiotherapy, and prognosis prediction.

Recently, deep learning has demonstrated great performance in medical image classification and recognition [14, 15]. Deep radiomic features have been extracted from CT images and illustrated an in-depth characterization of LN phenotypes from another perspective, which could be used to improve the predictive performance of LNM.[16, 17]. Deep learning models have also been applied to extract features to evaluate chemoradiotherapy treatment responses of ESCC in several studies [13]. Although these deep radiomic features improved the prediction of LNM compared with handcrafted radiomic features, how to explain the lesion representation from texture and size remains to be studied.

Some studies have shown that integrating clinical factors and radiomic features of tumors can improve the predictive performance of LNM in ESCC [4, 18–20]. These results indicate that radiomic features can sufficiently illustrate tumor heterogeneity and predict LNM in preoperative patients. However, most of the area under the curve (AUC) results were <0.8; therefore, improving the prediction of LNM using CT images is necessary. Few studies have directly analyzed the features of LNs in ESCC using deep radiomic features. In this study, we hypothesized that CT-based radiomic features of LN could be exploited to evaluate LN status in ESCC. Our aim was to analyze CT-based radiomic models that integrate the clinical, radiomic, and deep radiomic features of LNs to accelerate the predictive performance of LNM in patients with ESCC.

## 2. Materials and Methods

### 2.1. Data

Three hundred and fifty-five patients with ESCC who presented to Fuzhou First Hospital between February 2016 and December 2020 were enrolled in our study. The selection criteria were as follows: (a) underwent preoperative contrast-enhanced CT examination within 2 weeks, (b) confirmed ESCC histology, (c) confirmed LN status in pathology after surgery, and (d) complete clinical and pathological information available. The exclusion criteria were as follows: (a) patients whose LNs had poor-quality CT images (*n* = 6), (b) patients who received preoperative chemoradiotherapy (*n* = 34), (c) incomplete clinical and pathological information (*n* = 4), and (d) patients who presented with other malignancies (*n* = 3). The remaining 308 patients were randomly divided into two cohorts in a 7 : 3 ratio (training cohort, *n* = 216; test cohort, *n* = 92).

### 2.2. Image Acquisition and LN Segmentation

Patients underwent contrast-enhanced CT from the neck to the abdomen. The CT parameters are described in Section 1 of Appendix A1. As the location of the LNs was variable and complex, the maximum cross-sectional area of suspicious mediastinal LNs of one or several slices was selected for each patient according to previous reports [7]. The LNs as the region of interest (ROI) in each 2D image were automatically segmented according to the labels. These labels were delineated slice-by-slice by two experienced radiologists using the ITK-SNAP software (version 3.6.0, https://www.itksnap.org). Two radiologists assessed the segmentation results and reached a consensus through discussion. To evaluate the reproducibility of radiomics to segmentation uncertainty, we randomly chose CT images of 30 patients from the training cohort in an unknown manner. Radiomic features from the 2D ROI of LNs were extracted having intraclass correlation coefficients of >0.8.

### 2.3. Feature Extraction

**Figure 1:**
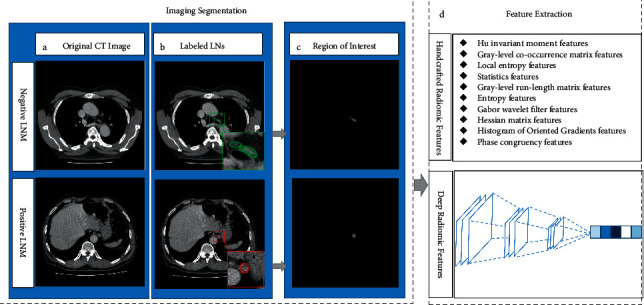
The extraction of radiomic features. (a) Original CT images with and without lymph node metastasis (LNM). (b) Labeled lymph nodes (LNs), negative LNM in the green box, positive LNM in the red box. (c) Region of interest (ROI) with and without LNM. (d) Feature extraction of handcrafted radiomic features and deep radiomic features.

To reduce the impact of various CT scanners, the DICOM images were uniformly converted to grayscale images during preprocessing. There may be only one or several lymph nodes in a slice, and according to the labels, the handcrafted radiomic features of LNs in all 2D images were automatically extracted for further analysis (Figure 1). The 207 features included 10 categories: (a) gray-levelco-occurrence matrix (GLCM); (b) gray-levelrun-length matrix (GLRLM); (c) Gabor wavelet filter (Gabor); (d) statistical features, including shape, size, and extremes; (e) histogram of oriented gradients (HOG); (f) local entropy; (g) Hu invariant moment (HU); (h) Hessian matrix (Hessian); (i) entropy; and (j) phase congruency (Phase). GLCM, GLRLM, Gabor, HOG, and local entropy are textural features, whereas HU, Phase, and Hessian features were the shape features. The handcrafted radiomic feature extraction process is detailed in Section 2 of Appendix A1. This process was implemented using MATLAB (version 2020a; MathWorks Inc., Natick, MA, USA).

Deep neural networks (DNNs) have already been successfully utilized to extract tumor features [13]. We extracted deep radiomic features using a pretrained ResNet50 model through transfer learning, according to the results of a previous study [21]. Global max pooling was used to select the maximum values of each layer in the feature maps as output values. Using the ResNet50 model, 1000 features were extracted from each patient. Deep radiomic feature extraction was implemented using Python software (version 3.7).

Clinical features extracted from the clinical and CT reports included patient age and sex.

### 2.4. Feature Selection

Dimensional reduction and extraction of optimal features were performed using the least absolute shrinkage and selection operator (LASSO) approach, as demonstrated in radiomic studies of LNM [8], in which a two-step feature selection method was applied. First, a *t*-test was used to reduce the dimensions of the features with *p* < 0.05. Then, the radiomic features were normalized using the standard scaler method. This preprocessing method ensured that the radiomic features were within similar ranges, which weakened the effect of radiomic features with different large discrete values. The LASSO method was utilized to choose the key features of these effective features. The hyperparameter “lamda” was optimized using 10-foldcross-validation with the smallest mean squared error. The key handcrafted radiomic and deep radiomic features were selected separately using the above method. The clinical features retained their original number. To implement feature selection, we used the scipy and sklearn packages (version 1.0) in Python software (version 3.7).

### 2.5. Radiomic Signatures and Predictive Models

To develop and validate the performance of multiple radiomic models that integrate clinical, handcrafted, and deep radiomic features, we constructed three combinations of three feature types after feature selection. First, a model consisting of handcrafted radiomic features (Model I) was constructed. Model II comprised deep radiomic features and handcrafted radiomic features. Model III consisted of clinical, handcrafted radiomic, and deep radiomic features.

These radiomic models were explored using machine learning methods, which included a support vector machine (SVM), adaptive boosting (AdaBoost), and random forest (RF) in the training cohort. We validated and evaluated the performance of the multiple radiomic models in both cohorts. Accuracy, specificity, sensitivity, area under the curve (AUC), and receiver operating characteristic (ROC) curves were obtained to determine the prediction performance of these radiomic models.

Meanwhile, for intelligent development in the future, we compared the best and worst models with the predictive results of the two radiologists and calculated and analyzed the AUC and ROC. These evaluation methods were implemented using Python software.

### 2.6. Statistical Analysis

Statistical analysis was performed using R software (version 3.5.3). The differences in clinical factors (categorical variables) between the two cohorts were computed using the chi-squared test. Categorical variables were presented as absolute numbers and percentages. Depending on the results of the normality tests, continuous variables were represented by either the mean ± standard deviation or the median and interquartile range. Independent sample *t*-tests or Wilcoxon tests were utilized to compare clinical factors (continuous variables) between cohorts with and without LNM. A two-sided *p* value <0.05 was considered statistically significant.

## 3. Results

### 3.1. Data Characteristics

In this study, 308 patients with ESCC were enrolled and divided into two cohorts (training cohort, *n* = 216; test cohort, *n* = 92). The clinical characteristics of the patients with and without LNM are listed in Table 1. No significant differences between cohorts were observed.

### 3.2. Feature Extraction and Selection

**Figure 2:**
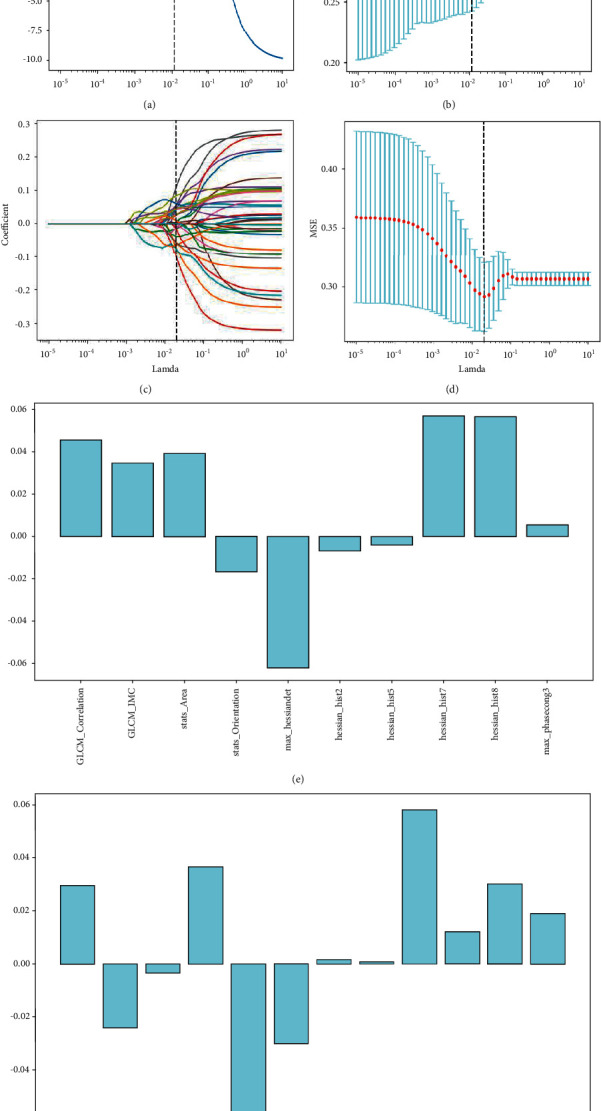
Selection of radiomic features and deep radiomic features associated with lymph node metastasis via LASSO method. (a) The coefficient profiles of 207 handcrafted radiomic features against the deviance explained. (b) The 10-foldcross-validation curve of handcrafted radiomic features with the optimal lamda value of 0.01151 and 10 nonzero coefficients. (c) The coefficient profiles of 1000 deep radiomic features against the deviance explained. (d) The 10-foldcross-validation curve of deep radiomic features with the optimal lamda value of 0.02024 and 12 nonzero coefficients. (e) The coefficient values of key handcrafted radiomic features. (f) The coefficient values of key deep radiomic features.

Eighteen of 207 extracted effective radiomic features had a *p* value <0.05, as determined by the *t*-test. Ten key handcrafted radiomic features were selected from the 18 effective features using LASSO with 10-foldcross-validation. Radiomic features are presented in Table 2. Equally, 40 of 1000 extracted effective deep radiomic features had a *p* value <0.05, after the *t*-test. Twelve key deep radiomic features were selected from the 40 effective features using LASSO with 10-foldcross-validation. The key deep radiomic features and their *p* values are listed in Section 3 of Appendix A1. According to the coefficient profiles, we selected key features with nonzero coefficients through 1000 iterations. Selected key lambdas were 0.01151 and 0.2024, respectively. These handcrafted radiomic features and deep radiomic feature reduction using LASSO are shown in Figure 2. After LASSO processing, we obtained the nonzero coefficients of the features. The coefficients of the Hessian features were larger than those of other key radiomic features, as illustrated in Figure 2.

### 3.3. Construction of Multiple Radiomic Models and Assessment

**Figure 3:**
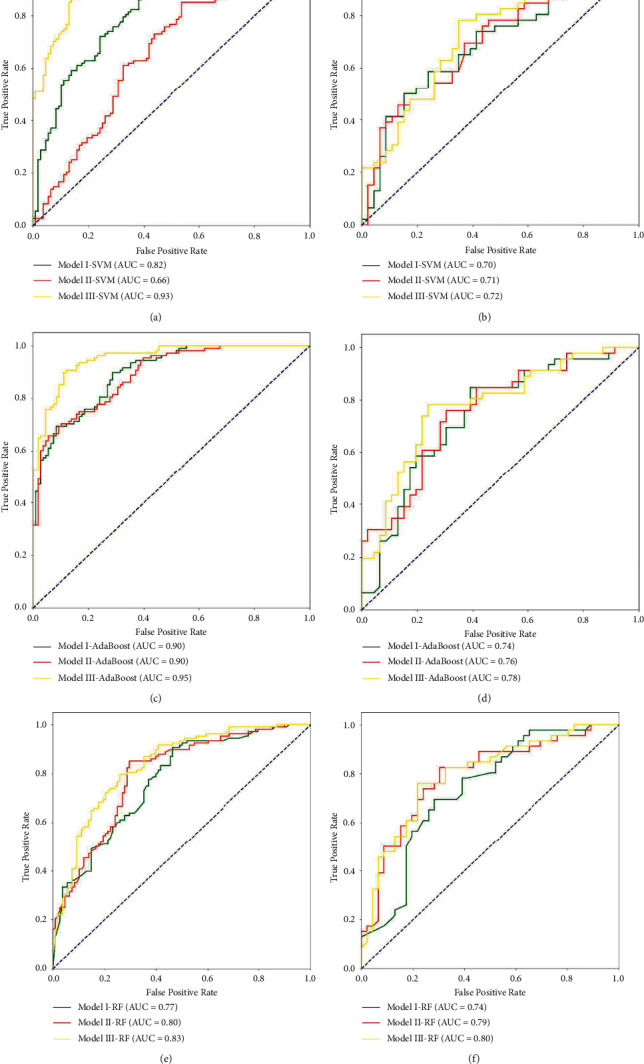
The receiver operating characteristic curves of the multiple models in the training and the test cohorts. (a) The ROC curves showing the predictive performances of the three models based on SVM in the training cohort. (b) The ROC curves showing the predictive performances of the three models based on SVM in the test cohort. (c) The ROC curves showing the predictive performances of the three models based on AdaBoost in the training cohort. (d) The ROC curves showing the predictive performances of the three models based on AdaBoost in the test cohort. (e) The ROC curves showing the predictive performances of the three models based on RF in the training cohort. (f) The ROC curves showing the predictive performances of the three models based on RF in the test cohort.

Key radiomic features were implemented to generate radiomic models to distinguish between patients with and without LNM. We constructed and assessed three radiomic models to select the best model; their performances, including accuracy, specificity, sensitivity, and AUC values, are shown in Table 3. The three models based on SVM, AdaBoost, and RF showed predictive performances with AUC values ranging from 0.66 to 0.95 in the training cohort and from 0.70 to 0.80 in the test cohort. We found that the AUC value of Model III was superior to those of Models I and II in the training and test cohorts, indicating that the combination of multiple features could improve prediction. The three models based on SVM performed worse than those based on RF and AdaBoost. The models based on RF showed better stability in the two cohorts, unlike those based on SVM and AdaBoost.  Figure 3 shows the ROC curves of these models for the training and testing cohorts.

**Figure 4:**
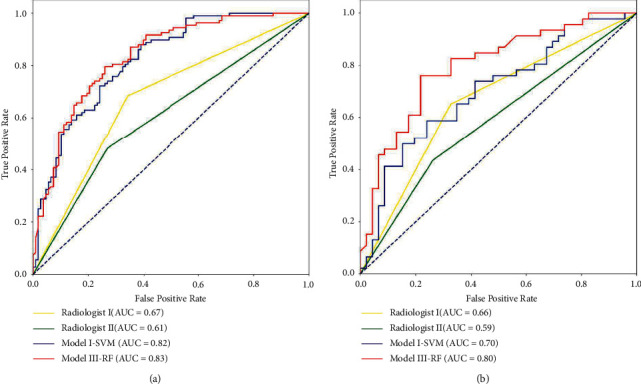
Comparison of the prediction of the models and the two radiologists in the training and test cohorts. (a) The ROC curves showing the predictive performances of the best model and worse model and two radiologists in the training cohort. (b) The ROC curves showing the predictive performances of the best model and worse model and two radiologists in the test cohort.

The comparative performance of the best radiomic model and that of the two radiologists is illustrated in Table 4. The ROC curves of the comparisons are shown in Figure 4. We found that the predictive performance of the best model was better than that of the two radiologists, and the quantitative analysis of radiomic features may help improve the prediction of LNM.

## 4. Discussion

In this study, we constructed three CT-based combined radiomic models to predict LNM in ESCC patients. Models I (handcrafted radiomic features), II (Model I plus deep radiomic features), and III (Model II plus clinical features) were constructed based on SVM, AdaBoost, and RF. Our results showed that these radiomic models, particularly Model III, have the potential to predict LNM in patients with ESCC based on RF. This could help clinicians estimate LN status for personalized chemoradiotherapy treatment and prognosis prediction.

In many previous studies, handcrafted radiomic features of CT images were based on the image biomarker standardization initiative (IBSI) [5, 13]. The key handcrafted radiomic features can improve the prediction model and prevent overfitting. These radiomic features describe the texture, size, and heterogeneity of LNs. Our results showed that GLCM, statistical, and Hessian features were selected through LASSO, which represented the complex texture and variable size of LN. Qu et al. indicated that GLCM features were associated with LNM in ESCC [4], and Piazzese et al. analyzed 2D GLCM features as corresponding stable features in esophageal cancer [11]. Our results were consistent with the findings of previous studies. Few studies have reported on the application of phase congruency in radiomics. Phase congruency provides reliable texture information under different illumination conditions [22]. Phase congruency is commonly used in palmprint authentication, image segmentation, face representation, and other tasks [22–26]. In our study, phase congruency was one of the key selected handcrafted radiomic features, indicating that it has a better representation of the texture complexity of LNs. Thus, it may be a representative radiomic signature for CT images and may contribute to the diagnosis and evaluation of other diseases.

In our study, models based on a combination of multiple features demonstrated the potential predictive performance of LNM. Our results showed that Models II and III were better than Model I, indicating that a combination of multiple types of features could enhance the prediction performance of LNM. This also indicated that the power of the deep learning method to determine the complex heterogeneity of LNs was better than expected. Furthermore, we used machine learning methods to construct nine radiomic models. Model III based on RF showed good stability and excellent prediction performance compared to other models based on SVM and AdaBoost in both cohorts. The results indicated the excellent generalization performance of the RF algorithm on the prediction model, particularly based on the combination of the three types of features. Many previous reports have focused on the radiomic features of the tumor, which were applied to predict LNM [5, 18, 19]. Indeed, Wu et al. proposed a multilevel CT radiomic model of tumors in ESCC; the AUC value of the predictive performance of LNM was 0.728 [18]. Shen et al. built a radiomics-based nomogram of tumors for the prediction of preoperative LNM in EC with an AUC of 0.771 [5]. Tan et al. validated that the radiomic nomogram of tumors outperforms the size criteria of LN from CT-reported in discriminating LNM, AUC of radiomics nomogram was 0.772 [19]. The above analysis of the radiomic features based on the tumor was useful to identify the preoperative LN status, but in our study, Model III based on RF (AUC value = 0.8) was better than the above results. This indicated that radiomic analysis of LN could improve the prediction of LNM in ESCC, particularly the combination of deep radiomic, handcrafted, and clinical features.

Many studies have recommended that the quantitative radiomic features of medical images could reveal the biological information of LN, which potentially improves LNM prediction and prognosis [8, 9, 17]. Our study analyzed the radiomic features of LN and built models combining multiple types of features to predict LN metastasis. Furthermore, we found that the prediction of Model III was greater than the results of the above reports, with an AUC of 0.80. This validated that the radiomic features of LN were useful for discriminating LN status, especially with the addition of clinical features. In brief, the combination of handcrafted radiomic features, deep learning radiomic features, and clinical features may be able to illustrate information about LN from different levels.

Clinically, the assessment of LN status is primarily performed by radiologists based on the LN axis ratio and size criteria calculated from preoperative CT images; however, this is controversial and inaccurate. In our study, to validate the potential ability of radiomic models, we compared the radiomic models with the discrimination results of LNM provided by two experienced radiologists, without knowing the pathological reports. The results showed that the discrimination results by the two radiologists were unsatisfactory, with an accuracy of 0.66 and 0.59 and an AUC of 0.66 and 0.59, respectively, which were worse than those of the models based on multiple radiomic features in the test cohort. This indicates that the size cannot satisfactorily illustrate the LN status only from vision, like previous reports [6, 27]. This may also affect the treatment and prognosis prediction of nonsurgical patients.

This study has several limitations. First, we only used single-center data for model training and validation. Prospective external validation of radiomic models with multicenter trials is required to generalize the results. Second, because the location of LN was complex and variable, we considered only the largest cross-sectional area of LN in each patient in this study, which may have impacted the last prediction because of incomplete LN information. A combination of radiomic analysis of the primary tumor and LNs with other omics techniques may potentially provide accurate prediction performance. Third, we collected contrast-enhanced CT images; however, some patients were unable to undergo this examination because of poor renal function or allergies. Compared with contrast-enhanced CT scan examination, non-contrast-enhanced CT scan examination is quicker and more convenient; therefore, we plan to investigate the radiomic features of non-contrast-enhanced CT images in the future.

## 5. Conclusions

In conclusion, we developed radiomic models combining handcrafted radiomic features with deep radiomic and clinical features, which could be clinically used to assess LN status in patients with ESCC. This could serve as an intelligent method for clinicians to estimate LN status for chemoradiotherapy and to facilitate prognosis prediction and preoperative assessment.

## Figures and Tables

**Table 1:** Demographic statistics of patients in the training cohort and test cohort.

Variable	Training cohort				Test cohort			
	LNM− (*n* = 108)	LNM+ (*n* = 108)	*χ* ^2^/*Z*	*p*	LNM− (*n* = 46)	LNM+ (*n* = 46)	*χ* ^2^/*Z*	*p*
Sex			4.256	0.039			2.841	0.092
Women	27	15			15	8		
Men	81	93			31	38		

Age			−0.543	0.587			−0.754	0.451
Mean	60.8	60.1			61.9	61.0		
Median	61.5	61			64.0	61.0		
Range	29.0∼82.0	37.0∼82.0			42.0∼85.0	45.0∼82.0		
SD	9.0	8.96			9.0	8.4		

LNM, lymph node metastasis; +, positive; −, negative; SD, standard deviation.

**Table 2:** Key radiomic features after LASSO.

Handcrafted radiomic category	Radiomic feature name	*p*_value
GLCM	GLCM_Correlation	0.010
	GLCM_IMC	0.031

Statistic	stats_Area	0.001
	stats_Orientation	0.038

Hessian	max_hessiandet	0.002
	hessian_hist2	0.024
	hessian_hist5	0.030
	hessian_hist7	0.040
	hessian_hist8	0.010

Phase congruency	max_phasecong3	0.045

*Note*. (1) Suffix of 2,5,7,8 mean the distribution histograms of the Hessian features. (2) Suffix of 3 means the different directions of the phase congruency. (3) IMC means information measure of correlation.

**Table 3:** The predictive performance of multiple radiomic models in the training and test cohorts.

Models	Training cohort				Test cohort			
	SEN	SPE	ACC	AUC	SEN	SPE	ACC	AUC
Model I-AdaBoost	0.81	0.73	0.77	0.90 (0.86, 0.94)	0.85	0.61	0.73	0.74 (0.64, 0.84)
Model II-AdaBoost	0.78	0.76	0.77	0.90 (0.85, 0.93)	0.78	0.61	0.70	0.76 (0.66, 0.86)
Model III-AdaBoost	0.91	0.86	0.88	0.95 (0.93, 0.98)	0.78	0.74	0.76	0.78 (0.69, 0.88)
Model I-SVM	0.80	0.67	0.73	0.82 (0.77, 0.88)	0.74	0.57	0.65	0.70 (0.60, 0.81)
Model II-SVM	0.63	0.61	0.62	0.66 (0.59, 0.73)	0.67	0.63	0.65	0.71 (0.61, 0.82)
Model III-SVM	0.89	0.81	0.85	0.93 (0.90, 0.96)	0.61	0.72	0.66	0.72 (0.62, 0.82)
Model I-RF	0.69	0.65	0.67	0.77 (0.71, 0.83)	0.70	0.70	0.70	0.74 (0.63, 0.84)
Model II-RF	0.75	0.72	0.74	0.80 (0.74, 0.86)	0.78	0.70	0.74	0.79 (0.70, 0.88)
Model III-RF	0.74	0.78	0.76	**0.83 (0.78, 0.89)**	0.76	0.76	0.76	**0.80 (0.71, 0.89)**

SEN, sensitivity; SPE, specificity; ACC, accuracy; AUC, area under the receiver operating characteristic curve; 95% confidence intervals are included in parentheses.

**Table 4:** The predictive performance of the best model and the two radiologists in the test cohort.

	Training cohort				Test cohort			
	SEN	SPE	ACC	AUC	SEN	SPE	ACC	AUC
Radiologist 1 (15 years of experience)	0.69	0.66	0.67	0.67	0.65	0.67	0.66	0.66
Radiologist 2 (5 years of experience)	0.48	0.73	0.61	0.61	0.43	0.74	0.59	0.59
Model III-RF	0.74	0.78	0.76	**0.83**	0.76	0.76	0.76	**0.80**

SEN, sensitivity; SPE, specificity; ACC, accuracy; AUC, area under the receiver operating characteristic curve.

## Data Availability

The datasets generated and/or analyzed during the current study are available from the corresponding author upon reasonable request.

## Abbreviations

AJCC: American Joint Committee on Cancer
AUC: Area under the curve
CT: Computed tomography
ESCC: Esophageal squamous cell carcinoma
GLCM: Gray-level co-occurrence matrix
GLRLM: Gray-level run-length matrix
LASSO: Least absolute shrinkage and selection operator
LN: Lymph node
LNM: Lymph node metastasis
ROC: Receiver operator characteristic
SVM: Support vector machine
RF: Random forest
AdaBoost: Adaptive boosting.

## Appendix

  Section 1: CT image acquisition parameters
  Section 2: radiomic features extracted
  Section 3: selected features after *t*-test and LASSO
